# Iatrogenic Acute Aortic Dissection in the Era of Minimally Invasive Cardiac Surgery - Experience of a Center and Review of Literature

**DOI:** 10.21470/1678-9741-2020-0561

**Published:** 2021

**Authors:** Daniele De Viti, Pierpaolo Dambruoso, Paolo Izzo, Ilir Dhojniku, Pasquale Raimondo, Carmine Carbone, Domenico Paparella

**Affiliations:** 1 Department of Cardiology, Santa Maria Hospital, GVM Care and Research, Bari, Italy.; 2 Department of Cardiac Anesthesia and Intensive Care, Santa Maria Hospital, GVM Care and Research, Bari, Italy.; 3 Department of Emergency and Organ Transplant, University of Bari "Aldo Moro", Bari, Italy.; 4 Department of Cardiac Surgery, Santa Maria Hospital, GVM Care and Research, Bari, Italy.; 5 Department of Medical and Surgical Sciences, University of Foggia, Foggia, Italy.

**Keywords:** Iatrogenic Diseases, Aneurysm, Dissecting, Mitral Valve, Cardiac Surgical Procedures, Femoral Artery, Treatment Outcome

## Abstract

**Introduction:**

Iatrogenic acute aortic dissection (IAAD) type A is a rare but potentially fatal complication of cardiac surgery.

**Methods:**

The purpose of this article is to review the literature since the first reports of IAAD in 1978, examining its clinical characteristics and describing operative details and surgical outcomes. Moreover, we reviewed the recent literature to identify current trends and risk factors for IAAD in minimally invasive cardiac surgery procedures, often related to femoral artery cannulation for retrograde perfusion.

**Results:**

We found that IAAD ranges from 0.04 to 0.29% of cardiac patients in overall trials and ranged from 0.12 to 0.16% between 1978-1990, before the minimally invasive surgical era. And we concluded that since the first cases to the recent reports, the incidence of IAAD has not significantly changed. As minimally invasive procedures are on the rise, some authors think that the incidence of IAAD could increase in the future; we think that using all the precaution - such a strict monitoring of perfusion pressure throughout the intervention, avoiding extremely high jet pressures using vasodilators, repositioning of arterial cannula, or splitting perfusion in both femoral arteries -, this complication can be extremely reduced. Finally, we describe a very singular case occurring during mitral valve replacement followed by spontaneous dissection of left anterior descending artery one month later.

**Conclusion:**

The present article adds to the literature a more detailed clinical picture of this entity, including patients' characteristics, the mechanism, timing, and localization of the tear, and mortality details.

**Table:** 

Abbreviations, acronyms & symbols				
AAR	= Ascending aorta replacement		MICS-FC	= Minimally invasive cardiac surgery with femoral cannulation
AD	= Aortic dissection			
AVR	= Aortic valve replacement		MIDCAB	= Minimally invasive direct coronary artery bypass
CABG	= Coronary artery bypass grafting		MIMVR	= Minimally invasive mitral valve replacement
CPB	= Cardiopulmonary bypass		MVP	= Mitral valvuloplasty
ECC	= Extracorporeal circulation		MVR	= Mitral valve replacement
GARY	= German Aortic Valve Registry		OLT	= One lung transplant
HT	= Heart transplant		OPCAB	= Off-pump coronary artery bypass
IAAD	= Iatrogenic acute aortic dissection		PARTNER	= Placement of Aortic Transcatheter Valve
LAD	= Left anterior descending coronary artery		STS	= Society of Thoracic Surgeons
LVA	= Left ventricular aneurysmectomy		TAVR	= Transcatheter aortic valve replacements
MIAVR	= Minimally invasive aortic valve replacement		TVR	= Tricuspid valve replacement

## INTRODUCTION

Iatrogenic acute aortic dissection (IAAD) type A is a rare but potentially fatal complication of cardiac surgery. Delayed diagnosis and treatment can lead to extremely high mortality rate^[1]^. Dissection can occur as a result of direct mechanical damage at the site of cannulation or clamping of the aorta, or at the site of proximal anastomosis. Some studies report that minimally invasive cardiac procedures with femoral cannulation are associated with IAAD^[1,2]^. As minimally invasive procedures are on the rise, the incidence of IAAD could increase in the future. We conducted this study reviewing our own cases in the last three years and describing one case of IAAD. As previous studies are limited by their small sample sizes and by the rarity of this entity, the purpose of the current paper is to review the literature since the first report of IAAD in 1978, examining the clinical characteristics and describing operative details, management, and surgical outcomes. Moreover, we reviewed the recent literature to identify current trends and risk factors for IAAD in minimally invasive cardiac surgery procedures.

## METHODS

Based on the time of presentation of aortic dissection (AD), we included in the definition of IAAD the AD developed intraoperatively and between the 1^st^ and 35^th^ postoperative days. Among 1,499 cardiac surgical procedures in our institution, from January 2017 to December 2019, we found one patient (0.06%) with IAAD. The retrospective review of literature was done searching in PubMed, Cochrane Library, Medical Literature Analysis and Retrieval System Online (or MEDLINE), Embase^®^, Google Scholar, and Scopus randomized trials of IAAD occurring as a complication of open-heart surgical procedures, including "intraoperative aortic dissection", "iatrogenic aortic dissection", and "perioperative aortic dissection" in the research. We selected studies in which demographic and clinical characteristics of the patients and data concerning original surgical procedures, including description of AD as well as rate and cause of mortality, were reported. Data are shown as media plus standard deviation. We excluded cases of late IAAD in previous cardiac surgery and studies in which IAAD occurred during transcatheter interventions.

We then extended our research with a second analysis to identify only trials of minimally invasive surgical interventions, including minimally invasive surgical access (no conventional sternotomy), all the off-pump coronary artery bypass grafting (CABG) interventions (also with sternotomy), and transcatheter aortic valve replacements (TAVR), that included information on arterial cannulation site, in which the rate of IAAD was reported, with the same purpose to detect demographic and clinical characteristics of the patients and data concerning surgical procedures, including description of AD as well as rate and cause of mortality.

### Case Report

In this peculiar case, a 59-year-old woman with dyspnea, severe mitral regurgitation, and atrial fibrillation was scheduled for elective mitral valve replacement (MVR). Coronary angiography showed mild atherosclerosis of coronary vessels without significant lesions. The right femoral artery was cannulated directly with a 19 French cannula (Medtronic DLP femoral) because right mini-thoracotomy access to the mitral valve was planned. After the opening of the chest, a bluish color of ascending aorta was noticed, and a type A AD was suspected, which was confirmed by transesophageal echocardiography. Management of AD with retrograde extension from entry tear in the descending aorta is controversial, especially when the false lumen of the ascending aorta is completely thrombosed in patients who are often clinically stable. In selected patients, a more conservative approach consisting of initial medical management with timely surgical repair gave excellent outcomes^[3]^.

Therefore, the surgery was continued converting the procedure in median sternotomy, the right subclavian artery was directly cannulated, and antegrade extracorporeal circulation (ECC) was initiated with good flow and pressures in the cannula maintaining a deep hypothermia until 24°C. MVR with a mechanical Bicarbon mitral valve prosthesis (n. 29) was performed, and cardiopulmonary bypass (CPB) was terminated without any significant event. In early postoperative period, a conservative medical approach based on hemodynamic stability of the patient was planned. A computed tomography scan performed in the 7^th^, 14^th^, and 19^th^ postoperative days revealed persisting type A acute AD with false lumen of ascending aorta without thrombosis. Based on this evidence, the surgical team changed the strategy towards a surgical approach. The patient successfully underwent surgery with a simple tube graft ascending aorta and hemiarch replacement and venous grafts to the right coronary artery because of suspected extension of dissection in right coronary sinus. The patient was discharged on the 15^th^ postoperative day with a regular postoperative course; her last echocardiography findings before discharge showed moderate left ventricular dysfunction (ejection fraction: 40%).

**Fig. 1:**
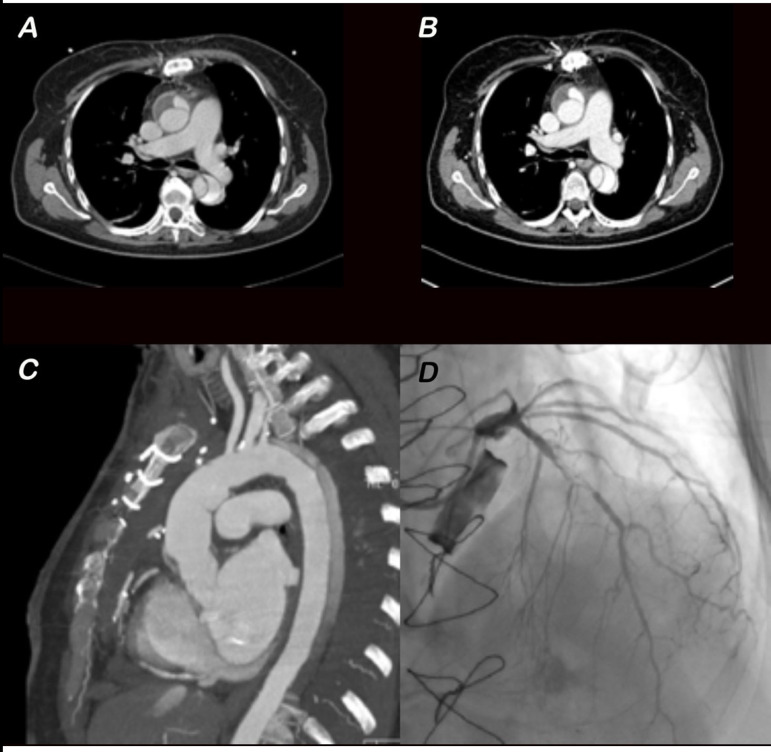
A) and B) Computed tomography imaging of iatrogenic acute aortic dissection in 7th and 19th postoperative days revealing persisting type A aortic dissection with false lumen without thrombosis in ascending aorta. C) Computed tomography performed after ascending aorta and hemiarch replacement. D) Angiography showing spontaneous dissection of left anterior descending coronary artery.

One month later, she was urgently readmitted for onset of acute dyspnea, chest pain, and worsening of ejection fraction rated with echocardiography. Evaluation by cardiac catheterization revealed spontaneous dissection of left anterior descending coronary artery (LAD), occlusion of right coronary artery, and venous graft. The patient was successfully treated with coronary angioplasty and stents on LAD (Figure 1), and a dual chamber implantable cardioverter defibrillator was implanted for primary prevention. She was discharged in good health, and the one-year follow-up was free of other important events.

## RESULTS

**Fig. 2:**
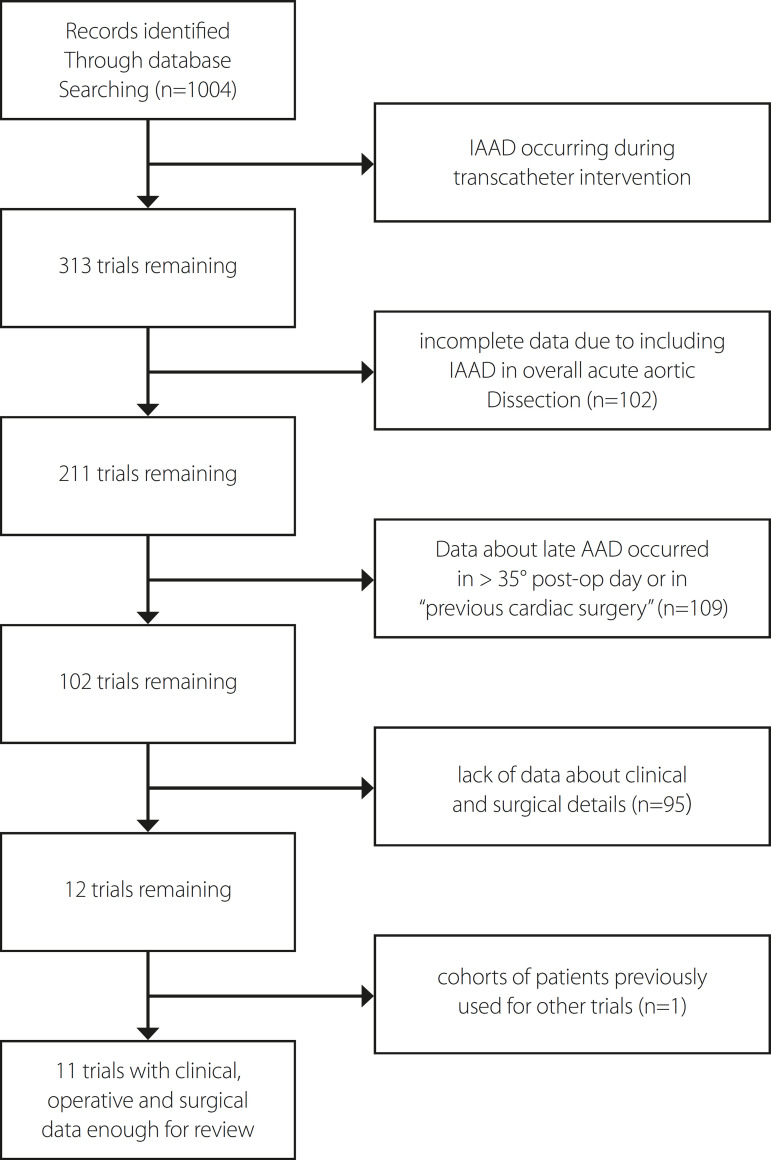
Consort flow diagram. We identified 1,004 records through database searching. Of these, only 11 trials were included for complete clinical, operative, and surgical data about iatrogenic acute aortic dissection (IAAD).

Almost 900 records were excluded immediately because of description of irrelevant data. After exclusion of these records, 102 papers were retrieved for full review. Eleven articles including relevant data on timing of AD, localization of intimal tear, surgical management, and rate and cause of mortality were included (Figure 2). IAAD ranged from 0.04 to 0.29% of cardiac patients (0.12-0.16% in the period of 1978-1990, before the minimally invasive surgical era). IAAD occurred intraoperatively in 70-100% of patients. The diagnosis was made during cannulation or after removing clamp in almost all cases, and the intimal tear was localized at the cannulation site in most of the patients. Findings are summarized in Tables 1 and 2.

**Table 1.** Clinical and anatomopathological data of patients with iatrogenic acute aortic dissection (IAAD) during cardiac surgery.

Trials	Ruchat	Still	Fleck	Ketenci	Polat	Shea	Hwang	Leontyev	Stanger	Williams	Abdusalom
**Original Procedure, n**											
	CABG, 5 Re-CABG, 1 AVR, 1 AVR + CABG, 1 MVP + CABG, 1 AAR, 1	CABG, 17 MVR + CABG, 2 AVR + CABG, 1 LVA + CABG, 1 LVA, 1 AVR, 1	CABG, 2 MVR, 3 AVR + AAR, 1 OLT, 1	CABG, 21	CABG, 4 AVR, 2 AVR + CABG, 1 MVR, 2 MVR + AVR, 1	AAR, 5 AVR, 4 CABG, 3 CABG + AVR, 2 HT, 1	MVR, 3 AVR, 1 Re-AVR, 1 TVR, 1 CABG, 2 AVR + CABG, 1 AAR, 1	MVR, 10 AVR, 10 CABG, 8 CABG + MVR, 3 CABG + AAR, 1 AVR + MVR, 1 Others, 3	CABG, 23 AVR, 5 AVR + CABG, 3 MVR, 2 MVR + CABG, 2 AVR + MVR, 1	CABG, 1053 AVR, 166 MVR, 75	OPCAB, 2
**Mean Age of IAAD Patients**											
Years	64 ±9	67	69.2 ±7.6		66.5±7	74 ±6.8	62.48.0		71.1 ±8.5	72 ±6	65 ±3
**Sex of Patients**											
Male/Female	07/03	14/10	03/04		05/05	05/09	04/06		20/16	795/499	01/01
**Hypertension**											
	8 (80%)	12 (50%)			6 (60%)	13 (87%)	3 (30%)		30 (83%)	995 (77%)	2 (100%)
**Anatomopathological Findings**											
Aortic Atherosclerosis	7 (70%)	5 (21%)	2 (28%)			5 (33%)	7 (70%)				2 (100%)
Aortic dilation		7 (29%)									

AAR=ascending aorta replacement; AVR=aortic valve replacement; CABG=coronary artery bypass grafting; HT=heart transplant; LVA=left ventricular aneurysmectomy; MVP=mitral valvuloplasty; MVR=mitral valve replacement; OLT=one lung transplant; OPCAB=off-pump coronary artery bypass; TVR=tricuspid valve replacement

**Table 2.** Surgical data of patients with iatrogenic acute aortic dissection (IAAD) during cardiac surgery.

Trials	Ruchat	Still	Fleck	Ketenci	Polat	Shea	Hwang	Leontyev	Stanger	Williams	Abdusalom
Patients, n	8,624	14,877	3	10,13	29,683	23,27	3,421	55,279	68,249	2,219,991	711
IAAD, n	10 (0.12%)	24 (0.16%)	7 (0.23%)*	21 (0.20%)	10 (0.04%)*	15 (0.06%)	10 (0.29%)*	36 (0.06%)	36 (0.05%)	1,294 (0.06%)	2 (0.28%)
**IAAD**											
Intraoperatively	7 (70%)	20 (83%)	7 (100%)	19 (90%)	10 (100%)	14 (93%)	10 (100%)	31 (86%)			2 (100%)
**Timing of Diagnosis**											
After aortic declamping		17		16	2	1	9				2
During aortic cannulation		1 (5%)		3 (16%)							
During aortic decannulation		2 (10%)			5 (50%)						
During insertion of cardioplegia cannula						11 (79%)					
Chest closure						1 (7%)					
During aortotomy						1 (7%)	1 (10%)				
During anastomosis	6 (83%)		4 (57%)		3 (30%)						
After the start of CPB			3 (43%)								
At the end of operation	1 (17%)										
**Site of Tear**											
Proximal anastomosis	2 (20%)	1 (4%)	7 (100%)		3 (30%)	1 (7%)	9 (90%)	2 (6%)	13 (36%)		2 (100%)
Cannulation	7 (70%)	10 (42%)			4 (40%)	13 (86%)		14 (39%)	7 (19%)		
Aortotomy	1 (10%)	1 (4%)				1 (7%)	1 (10%)	6 (17%)	2 (6%)		
Clamp		12 (50%)			3 (30%)			7 (19%)	8 (22%)		
Cardioplegia								7 (19%)	5 (14%)		
Unknown									1 (3%)		
**Type of Repair**											
Primary repair	1 (10%)	13 (54%)	7 (100%)		9 (90%)	15 (100%)	1 (10%)	36 (100%)			1 (50%)
Dacron graft	8 (80%)	7 (29%)			1 (10%)		9 (90%)				1 (50%)
Dacron patch	1 (10%)	4 (17%)									

* Only intraoperative IAAD were analyzed

CPB=cardiopulmonary bypass

The second research extended only to trials of minimally invasive surgical intervention, and TAVR was performed in 18 trials in which the rate of IAAD was reported. Unfortunately, data on demographic and clinical characteristics of the patients, as well as data concerning surgical procedures, description of AD, and rate and cause of mortality were not reported in these trials. The rate of IAAD in series of only minimally invasive surgical intervention and TAVR ranged from 0 to 1.5% (Table 3).

**Table 3.** Time and causes of death of patients with iatrogenic acute aortic dissection occurring during cardiac surgery.

Trials	Ruchat	Still	Fleck	Ketenci	Polat	Shea	Hwang	Leontyev	Stanger	Williams	Abdusalom
Mortality	4 (40%)	6 (25%)	3 (43%)	10 (48%)	3 (30%)	1 (7%)	4 (40%)	14 (39%)	9 (25%)	615 (48%)	2 (100%)
**Time of Death**											
Intraoperatively	1 (25%)	3 (50%)	0 (0%)	7 (70%)	0 (0%)	0 (0%)	0 (0%)	-	-	-	1 (50%)
Postoperatively	2 (50%)	3 (50%)	3 (100%)	3 (30%)	3 (100%)	1 (100%)	4 (100%)	-	-	-	1 (50%)
Death before surgery	1 (25%)	0	0	0	0	0	0	-	-	-	0
**Causes of Death**											
Decompensated heart failure/multi-organ failure	2 (100%)	2 (67%)	1 (33%)		2 (67%)	1 (100%)	3 (75%)		5 (56%)		1 (50%)
Respiratory insufficiency		1 (33%)	1 (33%)		1 (33%)		1 (25%)		2 (22%)		1 (50%)
Hypoxic brain damage			1 (33%)						2 (22%)		

## DISCUSSION

Our review considered not only IAAD, but also dissections which occurred hours and days after cardiac surgery. In our series, the incidence of this complication was 0.06%, according to the data from the reviewed literature in which the incidence of IAAD ranges from 0.04 to 0.29%. Since the first cases to the recent reports in the era of minimally invasive surgery the incidence of IADD has not significantly changed^[4]^.

There are important differences in clinical presentation, surgical management, and prognosis that have not been thoroughly discussed in the literature because of limited patient sample sizes. The present study adds to the literature a more detailed clinical picture of this entity, including patient characteristics, the mechanism, timing, and localization of the tear, and mortality details.

In most cases, predisposing factors such as history of hypertension, atherosclerosis of the aorta, ascending aortic aneurysm, or cystic medial necrosis can be identified^[5]^. Uncontrolled arterial hypertension is the main cause of the onset of damage to the intima of the aorta.

Since the first report of IAAD associated with open-heart surgery, most cases described are related to aortic cannulation or aortic cross-clamping^[6]^. In the case reported in our casuistry, IAAD was due to femoral cannulation. Femoral cannulation during CPB has become a common approach for many cardiac procedures and serves as an important access option, especially during minimally invasive cardiac surgery. Retrograde AD from femoral artery cannulation is another mechanism for aortic injury and occurs in as high as 3% of patients^[7]^. Using the Society of Thoracic Surgeons (STS) database, Williams et al.^[1]^ analyzed 2,2 million cardiac procedures from 1996 to 2007 and found that the femoral cannulation site was associated with IAAD. Conversely, Lamelas reported a casuistry of 2,400 minimally invasive cardiac surgical procedures utilizing femoral cannulation technique in which no AD occurred^[8]^. The analysis of the STS Adult Cardiac Surgical Database between 2004 and 2008 showed 0.09% rates of IAAD in a large cohort of 4,322 patients undergoing mitral valve operations performed with femoral, arterial and venous cannulation compared to 0.03% in conventional surgery^[9]^. It should be recognized that the femoral cannulation most commonly represents an alternative arterial cannulation site if IAAD occurs, and thus, it is not defined if the association with femoral arterial cannulation represents a cause of AD or a result. However, when retrograde perfusion through the femoral artery is used, a strict monitoring of perfusion pressure throughout the operation is needed. Avoiding extremely high jet pressures from the femoral cannula is essential in preventing retrograde AD. Particularly when endoartic balloon catheter is used for aortic occlusion, there is a substantial risk of having high jet pressure at the exit of the cannula. When perfusion pressure exceed a safety value use of vasodilators, repositioning of arterial cannula or splitting arterial perfusion in both femoral arteries are recommended^[10]^. More data are required to better investigate IAAD as a complication of femoral cannulation.

Since the adoption of off-pump coronary artery bypass (OPCAB) surgery, numerous investigators have compared its results with those of on-pump coronary bypass surgery. Some reports have reported a higher incidence of early postoperative acute AD occurring in OPCAB than in on-pump bypass surgery^[11]^. In 2001, a Montreal Heart Institute team published its initial experience with OPCAB surgery in 308 patients and compared it with the results in 2,723 patients who underwent on-pump coronary artery bypass^[2]^. There was a greater frequency of IAAD in the OPCAB group, IAAD occurred in three patients among 308 operated on without ECC (0.97%) and one patient among 2,723 operated on under ECC (0.04%) (P<0.00001). The authors concluded that the risk of AD might be increased in OPCAB. In a consecutive series of 300 OPCAB operations performed in elderly population between 1996 and 1999, Demers et al.^[12]^ reported one case of IAAD (0.33%). Lahtinen reported a series of 68 patients undergoing OPCAB using the Symmetry Bypass System Aortic Connector in which one patient (1.5%) died of ascending AD^[13]^. However, in the large database of Buffolo from September 1981 to November 2004, 3,866 consecutive patients were revascularized without CPB and there were only two cases (0.05%) of IAAD^[14]^. Although OPCAB does not require cannulation and aortic cross-clamping, it has been hypothesized that application of a lateral clamp can increase the risk of dissection due to a pulsatile pattern of arterial pressure during the application of a side-biting clamp and the performance of the proximal anastomoses, even if the aortic pressure can be decreased temporarily during a side-clamp application^[15]^. In the large analysis of Williams, the overall rate of AD in OPCAB patients was 0.04%, compared with 0.06% for CABG patients^[1]^. Similarly, of the 4,784 OPCAB cases of Narayan database, only two (0.04%) developed intraoperative AD. Comparing off-pump with on-pump procedures, OPCAB was not a risk factor for AD^[16]^. As minimally invasive procedures are on the rise, the incidence of IAAD could increase. Minimally invasive aortic valve replacement (MIAVR) has shown to have advantages over conventional aortic valve replacement such as less bleeding, shorter duration of mechanical ventilation, and reduced intensive care unit and hospital stays. However, it is also known to have disadvantages such as longer CPB and aortic cross-clamping times and potential complications related to peripheral cannulation^[17]^. In a recent report, our group has demonstrated in a large multi-center population that MIAVR is associated with reduced hospital mortality without increased risk of IAAD^[18]^. Similarly, no IAAD was reported in the 232 cases of isolated aortic valve replacement with mini J-sternotomy approach from Bakir^[19]^ and in the 1,000 procedures (526 MIAVR performed in mini-sternotomy, plus 474 minimally invasive MVR with right mini-thoracotomy approach) from Mihaljevic^[20]^. Among the different areas of cardiac surgery, the minimally invasive approach has achieved particular popularity for mitral valve treatment. A 12-year experience from the East Carolina University and University of Pennsylvania reported a total of 1,178 patients having minimally invasive mitral valve surgery (941 [80%] mitral valve repair, 237 [20%] MVR) in which IAAD occurred in 11 patients (0.9%); moreover, the authors revealed that the incidence of AD was higher when endoartic balloon occlusion was applied, even though this difference was not statistically significant^[21]^. Conversely, Malvindi et al.^[10]^ compared transthoracic aortic clamping and endoartic balloon occlusion technique utilized in minimally invasive cardiac surgery for valve pathology and did not observe any IAAD in both groups. However, current evidence is of low quality, and larger, more methodologically rigorous randomized trials are required. Finally, IAAD can present in other minimally invasive cardiovascular procedures such as diagnostic and therapeutic coronary artery intervention, thoracic endovascular aortic repair, and TAVR. With the rapid increase in the number of TAVR cases around the world, the association between IAAD and TAVR is also emerging in the literature. A spectrum of aortic injuries can occur post-TAVR and include those contained to the aortic wall and those in the periaortic space to rupture. The clinical presentation and the management vary widely as IAAD after TAVR procedure is reported as part of the major vascular complications. The incidence ranges from 0.2% in the German Aortic Valve Registry (or GARY) (33 IAAD of 15,964 patients) to 2% in transapical TAVR cases in the review of Segesser^[22,23]^. In the Placement of Aortic Transcatheter Valve (or PARTNER) trial, there were three patients (0.7%) with IAAD out of 419 included in the trial^[24]^. Despite increased operator experience and evolution of the technique, complications involving the aorta and aortic valve annulus are potentially catastrophic and present unique management challenges in the TAVR population. Although the incidence of IAAD remains low, TAVR patients are usually very old and with multiple comorbidities, and for these reasons, strategies to prevent this complication are essential.

## CONCLUSION

IAAD is an unpredictable and often fatal complication of cardiac surgery. Increased age, high blood pressure, and atheromatous disease of the ascending aorta are significant risk factors for iatrogenic dissection. Although the overall incidence of IAAD remains low, due to the increasingly elderly population undergoing cardiac surgery, strategies to prevent this complication are essential. Some authors reported that the risk of AD might be increased in OPCAB and in procedures performed with femoral artery cannulation, but we think that using all the precaution such a strict monitoring of perfusion pressure throughout the intervention, avoiding extremely high jet pressures using vasodilators, repositioning of arterial cannula, or splitting arterial perfusion in both femoral arteries, this complication can be extremely reduced. Finally, though current evidence shows that the minimally invasive surgical procedures can be performed safely, more methodologically rigorous randomized trials are required.

**Table:** 

Authors' roles & responsibilities	
DDV	Substantial contributions to the conception of the work; drafting the work; final approval of the version to be published
PD	Substantial contributions to the conception of the work; drafting the work; final approval of the version to be published
PI	Final approval of the version to be published
ID	Final approval of the version to be published
PR	Final approval of the version to be published
CC	Final approval of the version to be published
DP	Substantial contributions to the conception of the work; drafting the work; final approval of the version to be published

## Figures and Tables

**Table 4.** Trials of minimally invasive surgical interventions and transcatheter aortic valve replacement (TAVR) reporting the rate of iatrogenic acute aortic dissection (IAAD).

Trial	Period	Number of patients	Procedure	IAAD
Lamelas	2009-2015	2,4	MICS-FC	0 (0%)
Gammie	2004-2008	4,322	MICS-FC	4 (0.09%)
Chavanon	1995-1997	308	OPCAB	3 (0.097%)
Demers	1996-1999	300	OPCAB	1 (0.33%)
Lathinen	2002-2004	67	OPCAB	1 (1,5%)
Buffalo	1981-2004	3,866	OPCAB (83 MIDCAB)	2 (0.05%)
Bakir	1997-2004	232	MIAVR	0 (0%)
Mihaljevic	1996-2003	526	MIAVR	0 (0%)
Modi	1996-2008	1,178	MIMVR	11 (0.9%)
GARY registry	2011-2013	15,964	TAVR	33 (0.2%)
PARTNER trial	2007-2009	419	TAVR	3 (0.7%)

GARY=German Aortic Valve Registry; MIAVR=minimally invasive aortic valve replacement; MICS-FC=minimally invasive cardiac surgery with femoral cannulation; MIDCAB=minimally invasive direct coronary artery bypass; MIMVR=minimally invasive mitral valve replacement; OPCAB=off pump coronary artery bypass; PARTNER=Placement of Aortic Transcatheter Valve
